# Acute Myocardial Infarction Is a Risk Factor for New Onset Diabetes in Patients with Coronary Artery Disease

**DOI:** 10.1371/journal.pone.0136354

**Published:** 2015-08-21

**Authors:** Chul Soo Park, Woo Baek Chung, Yun Seok Choi, Pum Joon Kim, Jong Min Lee, Ki-Hyun Baek, Hee Yeol Kim, Ki Dong Yoo, Ki-Ho Song, Wook Sung Chung, Ki Bae Seung, Man Young Lee, Hyuk-Sang Kwon

**Affiliations:** 1 Department of Internal Medicine, College of Medicine, The Catholic University of Korea, Seoul, Republic of Korea; 2 Cardiovascular Center and Cardiology Division, Yeouido St. Mary’s Hospital, The Catholic University of Korea, Seoul, Republic of Korea; 3 Cardiovascular Center and Cardiology Division, Seoul St. Mary’s Hospital, Catholic University of Korea, Seoul, Republic of Korea; 4 Cardiovascular Center and Cardiology Division, Uijeongbu St. Mary’s Hospital, The Catholic University of Korea, Uijeongbu, Republic of Korea; 5 Cardiovascular Center and Cardiology Division, Bucheon St. Mary’s Hospital, The Catholic University of Korea, Bucheon, Republic of Korea; 6 Cardiovascular Center and Cardiology Division, St. Vincent Hospital, The Catholic University of Korea, Suwon, Republic of Korea; 7 Division of Endocrinology and Metabolism, Department of Internal Medicine, Yeouido St. Mary’s Hospital, The Catholic University of Korea, Seoul, Republic of Korea; Universitätsklinikum des Saarlandes, GERMANY

## Abstract

**Objective:**

To test the hypothesis that acute myocardial infarction (AMI) might accelerate development of new onset diabetes in patients with coronary artery disease independent of known risk factors.

**Methods:**

We conducted a retrospective cohort study within COACT (CathOlic medical center percutAneous Coronary inTervention) registry. From a total of 9,127 subjects, 2,036 subjects were diabetes naïve and followed up for at least one year with both index and follow-up laboratory data about diabetes. Cox proportional hazard model was used to derive hazard ratios (HRs) and 95% confidence interval (CI) for new onset diabetes associated with AMI in univariate and multivariate analysis after adjusting several covariates.

**Results:**

The overall hazard for diabetes was higher in AMI compared to non-AMI patients (p by log rank <0.01) with HR of 1.78 and 95% CI of 1.37–2.32 in univariate analysis. This association remained significant after adjusting covariates (HR, 1.54; 95% CI, 1.14–2.07; p<0.01). AMI was an independent predictor for higher quartile of WBC count in multivariate ordinal logistic regression analysis (OR, 6.75; 95% CI, 5.53–8.22, p<0.01). In subgroup analysis, the diabetogenic effect of AMI was more prominent in the subgroup without MetS compared to MetS patients (p for interaction<0.05). Compared to the reference group of non-AMI+nonMetS, the group of AMI+non-MetS (HR, 2.44; 95% CI, 1.58–3.76), non-AMI+MetS (HR, 3.42; 95% CI, 2.34–4.98) and AMI+MetS (HR, 4.12; 95% CI, 2.67–6.36) showed higher HR after adjusting covariates. However, the hazard was not different between the non-AMI+MetS and AMI+non-MetS groups.

**Conclusions:**

AMI patients have a greater risk of new-onset diabetes when compared to non AMI patients, especially those with mild metabolic abnormalities.

## Introduction

Diabetes mellitus is an important risk factor for coronary heart disease [1], and it is associated with higher prevalence of coronary heart disease and an unfavorable prognosis [2]. While there are broad evidences that diabetes showed worse outcomes after coronary revascularization procedures [3–6], there is a paucity of data regarding the incidence and risk factors for developing new-onset diabetes in patients with coronary artery disease.

Although recent data have shown that cardiovascular drugs such as statins [7] and beta blockers [8] are related with diabetes development, it has become clear that systemic inflammation largely contributes to its development [9–11].

Since Dutta et al. [12] revealed that acute myocardial infarction (AMI) triggered system-wide activity of innate immune cells leading to acceleration of remote area atherosclerosis in an animal model, this finding has also been demonstrated in humans using ^18^F-FDG positron emission tomography [13, 14]. Furthermore, AMI accelerated non-culprit coronary lesion atherosclerosis [15]. Therefore, we hypothesized that after AMI, the patients might have higher risk of new-onset diabetes compared with non AMI patients by activated systemic inflammatory response.

## Materials and Methods

### Study Population and Assessment

We used the COACT (CathOlic medical center percutAneous Coronary inTervention) registry, a large observational registry of demographic, clinical, and procedural data, including clinical outcomes of “all-comer” patients who underwent percutaneous coronary intervention with drug eluting stent implantation at any of the eight affiliated hospitals of the Catholic University of Korea between January 2004 and December 2009. Of the 9,127 patients enrolled in this registry, 4,527 were diabetes naïve at index admission. Diabetes naïve was defined as a patient with no self-reported or documented history of diabetes and proved glycated hemoglobin (HbA1c) < 6.5% or fasting blood glucose (FBG) <126 mg/dl at index admission. The patients with previous revascularization, with previous MI, whose follow up duration was less than 1 year before censoring, whose laboratory data of HbA1c and FBG were missing, who were taking steroid, who were receiving dialysis, and cancer patients treated during the follow up, were excluded. Thus, the data of total 2,036 subjects were available for analysis (S1 Table). All of the laboratory and clinical parameter data were obtained from careful review of patients’ medical records at index admission. This study protocol complied with the principles of the Declaration of Helsinki (revised in 2000). The Institutional Review Board of Yeouido Saint Mary's Hospital, The Catholic University of Korea, Seoul, Korea approved this study (No. SC15RISI0005) and waived the need for informed consent because the data were analyzed anonymously.

### Study End Points and Definition

New-onset diabetes was our primary end point and it was diagnosed using the 2010 criteria of the American Diabetes Association [16]. According to this definition, subjects with FBG ≥ 126 mg/dl and/or HbA1c ≥ 6.5% and/or post-challenge glucose (glucose at 2 h after a 75 gm oral glucose load) ≥ 200 mg/dl measured during the follow-up period were diagnosed with new-onset diabetes. Date of onset was defined as the date when laboratory measurement was conducted.

Acute myocardial infarction was defined as a myocardial infarction including ST elevation myocardial infarction and non ST elevation myocardial infarction that had occurred within the four weeks prior to enrollment into the COACT registry. A definite ST-elevation myocardial infarction was diagnosed if typical electrocardiogram changes were present along with prolonged chest pain, refractory to sublingual nitrates and/or enzyme or troponin T elevations (>0.1 μg/L). Non ST-elevation myocardial infarction was diagnosed, if symptoms and/or troponin T criteria, but not the ECG criteria for ST-elevation myocardial infarction were met. The modified National Cholesterol Education Program Adult Treatment Panel III (NCEP-ATP III) was used for the definition of metabolic syndrome (MetS) [17]. Because in most of the subjects, waist circumference was not measured, we defined obesity as body mass index (BMI) ≥25 kg/m^2^ as suggested by the position statement of the American College of Endocrinology [18]. Therefore, MetS was defined by the presence of three or more of the following risk factors: (1) obesity, BMI ≥25 kg/m^2^; (2) high triglycerides, ≥150 mg/dL; (3) low high density lipoprotein cholesterol (HDL-C), <50 mg/dL in women and <40 mg/dL in men; 4) increased systolic blood pressure, ≥130 mmHg or increased diastolic blood pressure, ≥85 mmHg or taking antihypertensives, and (5) high fasting plasma glucose, ≥100 mg/dL.

### Statistical Analysis

Continuous variables were expressed as mean±SD and the differences between groups were analyzed by Student’s *t-*test. Categorical variables were expressed as frequencies and percentage and analyzed by chi-squared test. Incidence rate of diabetes was calculated by the number of new-onset diabetes cases divided by person year at risk. We constructed Kaplan Meier cumulative hazard curve for diabetes incidence for the non-AMI and AMI group and compared the curves with log rank test. Individual risk factors were evaluated with univariate Cox proportional hazard analysis and multivariate Cox proportional hazard model was fitted using important variables showing significant association with new-onset diabetes in univariate analysis. To investigate the association between systemic inflammation and AMI, we analyzed the association between quartile of WBC count and risk factors using ordinal logistic regression analysis. Subgroup analysis was performed to estimate the absence or presence of interaction between AMI and other variables on diabetes incidence. We also evaluated the risk of diabetes among groups divided according to the AMI and metS status which includes the group of non AMI with (non-AMI+MetS) and without MetS (non-AMI+non-MetS) and the group of AMI with (AMI+MetS) and without MetS (AMI+non-MetS) by multivariate Cox proportional hazard model. A two-tailed p value of ≤0.05 was considered statistically significant. Statistical analysis was carried out by SPSS 19.0 and R statistical language v. 2.15.0.

## Results

### Patients

The 2,036 patients in this study accumulated 11,610 person-years, with an average follow up of 5.7 ± 1.9 years per patients. Over the 10-year of study period, we observed 224 subjects (11.0%) of new diabetes and the incidence rate was 19 cases per 1,000 person-years. Clinical characteristics of AMI and non-AMI patients were described in Table 1. The AMI patients were younger and had lower BMI, higher FBG and higher LDL cholesterol and lower TG level, with less prevalence of hypertension than non-AMI patients. Thus, the proportion of MetS was not different between those two groups. Inflammatory markers including WBC count and hs-CRP level were elevated in AMI compared to non-AMI patients. Statin and beta blockers were more frequently used in AMI compared to non AMI patients.

**Table 1:** Clinical characteristics of patients at index admission.

Characteristics	Non-AMI	AMI	p value
	(n = 1354)	(n = 682)	
Demographic			
Age, year	62 ±10	58 ± 12	<0.01
Male, *n*	901 (67)	549 (81)	0.31
BMI, kg/m^2^	24.9 ± 3.1	24.4 ± 3.0	<0.01
Hypertension, *n*	761 (56)	290 (43)	0.63
Current smoker, *n*	160 (12)	79 (12)	0.71
MetS, *n*	490 (36)	235 (35)	<0.01
Diagnosis			
Stable angina, *n*	854 (63)	0 (0)	
Unstable angina, *n*	500 (37)	0 (0)	
NSTEMI, *n*	0 (0)	230 (34)	
STEMI, *n*	0 (0)	452 (66)	<0.01
Laboratory findings			
FBG, mg/dl	100 ± 13	111 ± 18	<0.01
WBC count, 10^3^/mm^3^	6.85 ± 2.06	10.09 ± 3.58	<0.01
Creatinine, mg/dl	0.98 ± 0.45	1.03 ± 0.35	0.19
Total cholesterol, mg/dl	180 ± 39	182 ± 40	<0.01
Triglyceride, mg/dl	143 ± 77	114 ± 68	<0.01
HDL cholesterol, mg/dl	43 ± 11	42 ± 10	<0.01
LDL cholesterol, mg/dl	109 ± 35	118 ± 36	<0.01
hs-CRP, mg/l	0.51 ± 1.73	1.73 ± 3.29	<0.01
Extent of CAD			
One vessel, *n*	681 (50)	332 (49)	
Two vessel, *n*	432 (32)	210 (31)	
Three vessel, *n*	241 (18)	140 (20)	0.12
LVEF, %	62 ± 8	55 ± 10	<0.01
Discharge medication			
Aspirin, *n*	1344 (99.3)	679 (99.7)	0.22
Clopidogrel, *n*	1354 (99.9)	782 (100)	0.73
Statin, *n*	1113 (82.6)	604 (88.6)	0.32
Beta blocker, *n*	897 (66.2)	519 (76.1)	<0.01
ACEI/ARB, *n*	1027 (75.8)	543 (79.6)	0.47
Mean follow-up, year	5.8 ± 1.9	5.6 ± 1.9	<0.01

Data are mean±SD and number (%) of patients. AMI, acute myocardial infarction; BMI, body mass index; MetS, metabolic syndrome, NSTEMI, non ST segment elevation myocardial infarction; STEMI, ST segment elevation myocardial infarction; FBG, fasting blood glucose; HDL, high density lipoprotein; LDL, low density lipoprotein; CAD, coronary artery disease; LVEF, left ventricular ejection fraction; ACEI, Angiotensin converting enzyme inhibitor; ARB, Angiotensin receptor blocker.

### Association of AMI with New-onset Diabetes

**Fig 1:**
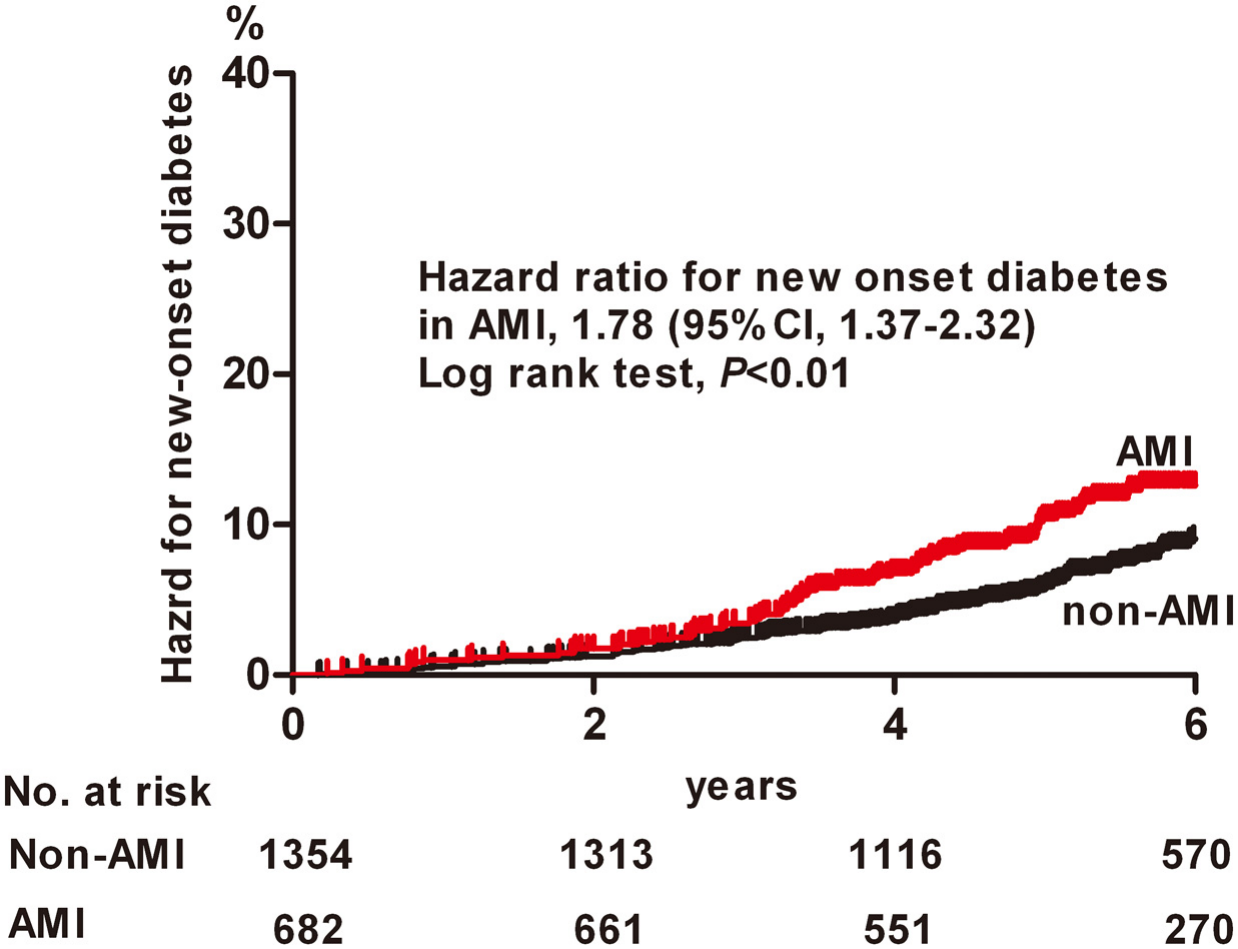
Kaplan-Meier curve for hazard for diabetes incidence in patients with AMI and non- AMI. Hazards were compared by log rank test. AMI, acute myocardial infarction.

Incidence rate of diabetes was 16 cases per 1000 person-years in non-AMI and 27 cases per 1,000 person years in AMI patients. As shown in Fig 1, the overall hazard for diabetes was significantly higher in the AMI compared to non AMI group (p by log rank <0.01) with hazard ratio (HR) 1.78 and 95% confidence interval (CI) 1.37–2.32. The probability of 5-yr hazard for diabetes was 5.9% (95% CI 4.5–7.2%) in non-AMI and 10.8% (95% CI 8.3%- 13.3%) in AMI patients. Mean diabetes free survival time was 9.3 years (95% CI 9.1–9.4 years) in non-AMI and 8.8 years (95% CI 8.5–8.9 years) in AMI patients. This association remained significant (HR, 1.54; 95% CI, 1.14–2.07; p<0.01) after adjusting other confounding factors including age<65 yrs, sex, MetS, WBC count, beta blocker use in multivariate Cox proportional hazard regression analysis (Table 2, Model 3).

**Table 2:** Univariate and multivariate Cox proportional hazard regression analysis for new-onset diabetes.

Variables	Model 1*			Model 2†			Model 3‡		
	HR	95% CI	p value	HR	95% CI	p value	HR	95% CI	p value
Age<65 yrs	1.32	1.00–1.75	0.05	1.13	0.84–1.52	0.42	1.14	0.85–1.53	0.39
Male	1.12	0.83–1.52	0.44	1.09	0.80–1.50	0.58	1.06	0.78–1.46	0.71
BMI≥ 25 kg/m^2^	1.79	1.37–2.33	<0.01						
Hypertension	1.14	0.88–1.49	0.32						
FBG≥ 100 mg/dl	3.31	2.54–4.31	<0.01						
Triglyceride≥ 150 mg/dl	1.55	1.18–2.01	<0.01						
HDL cholesterol	1.36	1.04–1.79	0.02						
< 50 in women									
< 40 in men									
MetS vs non-MetS	2.54	1.95–3.31	<0.01	2.51	1.92–3.28	<0.01	2.50	1.91–3.27	<0.01
WBC count, 10^3^/mm^3^	1.07	1.03–1.11	<0.01	1.06	1.02 1.11	<0.01	1.03	0.99–1.08	0.15
hs-CRP, mg/l	1.01	0.95–1.07	0.80						
AMI vs non AMI	1.78	1.37–2.32	<0.01				1.54	1.14–2.07	<0.01
LVEF<40%	1.43	0.76–2.70	0.27						
Beta blocker use	1.46	1.08–1.97	0.02	1.34	0.99–1.82	0.06	1.33	0.98–1.81	0.07
Statin use	1.44	0.98–2.12	0.06						
ACEI/ARB use	1.20	0.87–1.66	0.26						

*Univariate analysis.

^†^Model adjusted for age<65 yrs, sex, metabolic syndrome, WBC count and beta blocker use.

^‡^Model adjusted for AMI and variables of model 2.

BMI, body mass index; FBG, fasting blood glucose; HDL, high density lipoprotein; MetS, metabolic syndrome; AMI, acute myocardial infarction; LVEF, left ventricular ejection fraction; ACEI, Angiotensin converting enzyme inhibitor; ARB, Angiotensin receptor blocker.

### Association of Other Risk Factors with New-onset Diabetes

Univariate analysis revealed that, among the components of MetS, the parameters of BMI ≥25 kg/m^2^, FBS ≥100 mg/dl, triglyceride ≥150 mg/dl, HDL≤40 mg/dl in men and ≤ 50 mg/dl in women were associated with increased risk of diabetes. In addition, MetS, WBC count, beta blocker use were also associated with the development of diabetes. WBC count and MetS were associated with high risk of diabetes after adjusting age<65yrs, sex and beta blocker use (Table 2, Model 2). However in mulitivariate analysis after adding AMI as covariate, only MetS (HR, 2.50; 95% CI, 1.91–3.27; p<0.01) was independently associated with increased risk of the development of diabetes besides AMI (Table 2, Model 3).

### Association of AMI with Systemic Inflammation

We analyzed the impact of AMI on systemic inflammation which can be reflected by WBC count. In univariate ordinal logistic regression analysis, age<65 yrs, sex, hypertension, FBG≥ 100 mg/dl, LVEF< 40%, AMI, beta blocker and statin use were correlated with quartile of WBC count (Table 3, model 1). AMI was positively correlated with quartile of WBC count (odds ratio, 6.75; 95% CI, 5.53–8.22; p<0.01) after adjusting age<65yrs, sex, MetS, LVEF<40%, beta blocker use and statin use (Table 3, model 2).

**Table 3:** Univariate and multivariate ordinal logistic regression analysis for association between quartile of WBC count and risk factors.

Variables	Model 1*			Model 2†		
	OR	95% CI	p value	OR	95% CI	p value
Age<65 yrs	1.74	1.48–2.05	<0.01	1.48	1.23–1.76	<0.01
Male	1.91	1.60–2.72	<0.01	1.46	1.20–1.78	<0.01
BMI≥25 kg/m^2^	0.96	0.82–1.12	0.57			
Hypertension	0.65	0.55–0.76	<0.01			
FBG≥100 mg/dl	2.19	1.83–2.61	<0.01			
Triglyceride≥150 mg/dl	0.90	0.76–1.07	0.24			
HDL cholesterol	1.13	0.97–1.32	0.12			
< 50 in women						
< 40 in men						
MetS vs non-MetS	1.13	0.66–1.33	0.14	1.23	1.03–1.47	0.02
LVEF<40%	2.61	1.68–4.06	<0.01	1.63	1.03–2.59	0.04
AMI vs non-AMI	7.49	6.21–9.03	<0.01	6.74	5.53–8.22	<0.01
Beta blocker use	1.34	1.21–1.70	<0.01	1.21	1.01–1.46	0.04
Statin use	1.42	1.14–1.76	<0.01	1.26	0.999–1.56	0.051
ACEI/ARB use	0.86	0.72–1.04	0.12			

WBC count was categorized into 4-level ordinal scale defined by quartile.

*Univariate analysis.

^†^Model adjusted for age<65 yrs, sex, metabolic syndrome, LVEF<40%, AMI, statin use and beta blocker use.

BMI, body mass index; FBG, fasting blood glucose; HDL, high density lipoprotein; MetS, metabolic syndrome; LVEF, left ventricular ejection fraction; AMI, acute myocardial infarction; ACEI, Angiotensin converting enzyme inhibitor; ARB, Angiotensin receptor blocker.

### Subgroup Analysis

**Fig 2:**
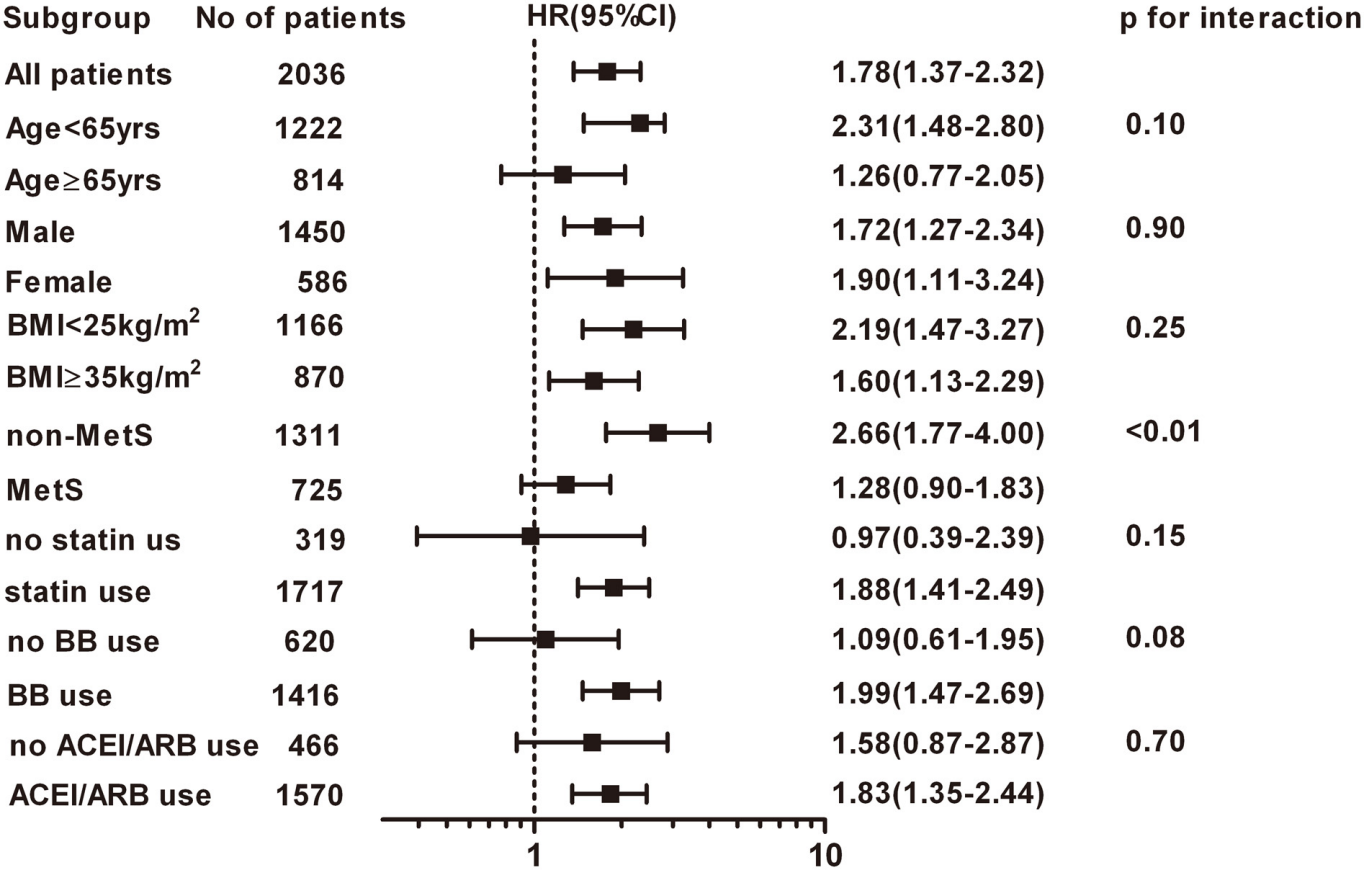
The influence of AMI in subgroups defined according to the various clinical factors. HR was calculated by Cox proportional hazard regression analysis. Interaction was estimated by the statistical significance of difference in HRs between groups. AMI, acute myocardial infarction; HR, hazard ratio.

We evaluated the influence of AMI on new-onset diabetes in subgroups divided by age, sex, BMI, MetS, statin use, beta blocker use, and ACEI/ARB use. Effect modification was observed in the subgroup divided according to the presence or absence of MetS. Diabetogenic effect of AMI was more 2 times prominent in non-MetS patients compared with MetS patients with HRs of 2.66 and 1.28, respectively (p for interaction <0.01, Fig 2).

### Combined Effect of AMI and Metabolic Syndrome on New-onset Diabetes

**Fig 3:**
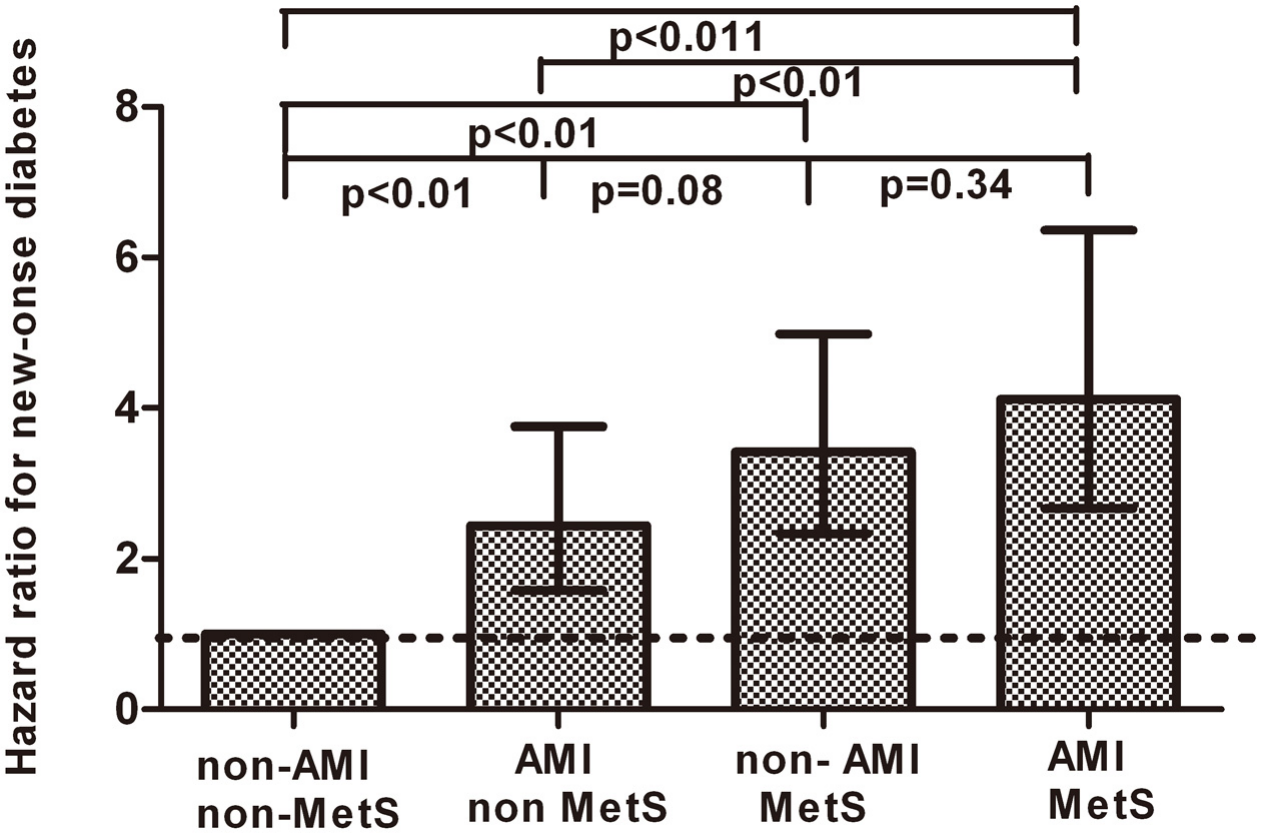
HRs of new-onset diabetes in patients defined by the presence or absence of AMI and MetS. Reference group was non-AMI+non-MetS. HRs were calculated using Cox proportional hazard regression analysis after adjustment for age < 65 yrs, male sex, quartile of WBC count and beta blocker use. I bars indicate 95% confidence interval. AMI, acute myocardial infarction; MetS, metabolic syndrome, HR, hazard ratio.

Compared to the group of non-AMI+non-MetS, the risk of diabetes was higher in the group of AMI+non-MetS (HR, 2.44; 95% CI, 1.58–3.76), non-AMI+MetS (HR, 3.42; 95% CI, 2.34–4.98), and AMI+MetS (HR, 4.12; 95% CI, 2.67–6.36) after adjusting age < 65 yrs, sex, quartile of WBC count and beta blocker use (all p<0.01). When compared to the non AMI+MetS group, the group of AMI+non-MetS showed similar risk (Fig 3).

## Discussion

We found that AMI accelerates the development of diabetes in patients with coronary artery disease. In particular, the diabetogenic effect of AMI is more prominent in non-MetS compared with MetS patients.

Development of diabetes is of multifactorial origin. Several studies [19–23] in the general population have now confirmed that the presence of inflammation predicts the development of diabetes. Obesity is the hallmark of metabolic syndrome and predisposes patients to the development of major chronic metabolic disease including type 2 diabetes. Despite the well- known link between obesity and diabetes, the mechanism remains elusive. It now appears that, in most obese patients, obesity is associated with a low-grade inflammation of white adipose tissue resulting from chronic activation of the innate immune system and subsequently it can lead to insulin resistance, impaired glucose tolerance and even diabetes [10, 24]. However, factors promoting the emergence of low grade systemic inflammation are probably complex. In this study, we found that obesity and MetS were strongly associated with diabetes development which is consistent with previous studies. However, in the current cohort of coronary artery disease patients, AMI was an independent predictor for new-onset diabetes even after adjusting other known risk factors including metabolic syndrome. Especially, the influence of AMI on new-onset diabetes was significantly highlighted in patients without MetS with HR of 2.66 compared with overt MetS patients with HR of 1.28. And the contribution of AMI on development of diabetes in patients without MetS was comparable with that of MetS in non-AMI patients. These findings suggest that even though MetS and obesity are powerful predictors for diabetes in general population, for the patients with coronary artery disease, AMI can be another important risk factor which exerted comparably with MetS. Furthermore, for the patients with mild metabolic abnormality, AMI could be the most powerful contributor for development of diabetes.

Patients surviving AMI have a higher risk of re-infarction and cardiovascular mortality compared with stable coronary artery disease patients [25, 26]. Several studies attempting to explain these findings demonstrated that AMI patients have multiple vulnerable plaques that can lead to future cardiovascular events [27–29]. However, AMI itself clearly accelerates atherosclerosis by infarct-triggering burst of systemic inflammation aimed at repair of injured heart. Dutta et al. [12] demonstrated that in Apoe-/- mice, after coronary artery ligation, the size of aortic plaques increased and vulnerable lesion morphology was induced with higher inflammatory cell content and protease activity, fuelled by persistently increased myeloid cell flux to atherosclerotic sites activated by heightened sympathetic nervous system activity. Recent studies [13, 14] also demonstrated in humans that ^18^F-FDG uptake increased in infarcted myocardium and it was correlated with uptake of remote myocardium, spleen and bone marrow. Besides, a correlation was found between spleen uptake and carotid artery uptake.

In our present study, the AMI patients showed increased WBC count and hs-CRP level compared to non AMI patients, suggesting increased systemic inflammation by AMI. Furthermore, a high WBC count was associated with higher risk of diabetes in univariate analysis, and it was an independent predictor for new-onset diabetes after adjusting age <65 years, sex, MetS and beta blocker use. However, this association disappeared after adjusting with AMI as a covariate in full model. This finding suggests that increased WBC count might accelerate diabetes development in association with AMI. To confirm this hypothesis, we analyzed the impact of AMI on increased WBC count. In multivariate ordinal logistic regression analysis, AMI was a most powerful independent predictor for increased WBC count with odds ratio of 6.75 after adjusting other risk factors. Because leukocytosis is a kind of acute inflammatory response, WBC count measured during index admission especially in AMI circumstances might not correlate with the degree of chronic systemic inflammation.

Taken together, a large body of epidemiologic data regarding association of inflammation and incident diabetes, recent human and animal research results suggesting burst of systemic inflammation by AMI and the present study support a reasonable hypothesis: Given systemic inflammation should precede diabetes development, for patients with coronary artery disease, myocardial necrosis induced by AMI might trigger acceleration of systemic inflammation leading to development of diabetes even in patients with mild metabolic abnormalities. To date, most of the research has focused on obesity, metabolic syndrome and cardiovascular drugs as etiologies of diabetes. However, based on our data, we can suggest a substantial role of systemic inflammation induced by various clinical events like AMI or chronic infection on diabetes incidence.

There have been conflicting data regarding the association between cardiovascular drugs and diabetes incidence. Statin treatment increased the risk of diabetes [30, 31], which was higher with intensive compared to moderate treatment [32]. In our study, we could not find a significant association between statin treatment and diabetes incidence which might be explained by the fact that the anti-inflammatory effect of statin outweighs the adverse effect on glucose metabolism in patients with overt coronary artery disease. However, given the fact that there was marginal association between statin use and diabetes and the adherence to statin during outpatient follow-up was not properly evaluated, the association still remains uncertain in patients with coronary artery disease. In contrast to statin, we found an association of beta blocker use with new-onset diabetes consistent with previous studies in univariate analysis [8, 33]; however, this association disappeared in multivariate analysis possibly due to more frequent use of beta blocker in AMI patients and the overwhelming diabetogenic effect of AMI compared with beta blocker use in multivariate analysis.

There are several limitations in our study.

First, the present study was conducted in a retrospective manner which might pose a quality problem of data at the time of enrollment. However, because patients with coronary artery disease usually have similar risk factors for diabetes, baseline clinical information was thoroughly obtained with careful interview and laboratory measurement. Second, the COACT registry was originally designed to evaluate the outcomes related with coronary events; hence, regular follow-up of FBG, HbA1c and 75 gm oral glucose loading test was not conducted, which might lead to underestimate the incidence of diabetes. Third, WBC count and hs-CRP concentration might not precisely reflect inflammatory status. It might be the reason why no strong correlation between WBC and development of diabetes was observed. Future studies using other inflammatory markers such as fibrinogen, TNFs, ILs, and adhesion molecules are warranted. In addition, serial follow-up of these inflammatory markers would be needed to investigate whether or not AMI affects systemic inflammation even after recovering from acute events.

In conclusion, AMI patients have a greater risk of new-onset diabetes when compared to non AMI patients, especially those with mild metabolic abnormalities. Even though, the clinical impact of new-onset diabetes in patients after PCI is uncertain, more rigorous follow-up is necessary in patients after AMI.

## Supporting Information

S1 Table Dataset of new-onset diabetes derived from COACT registry. (XLS)
